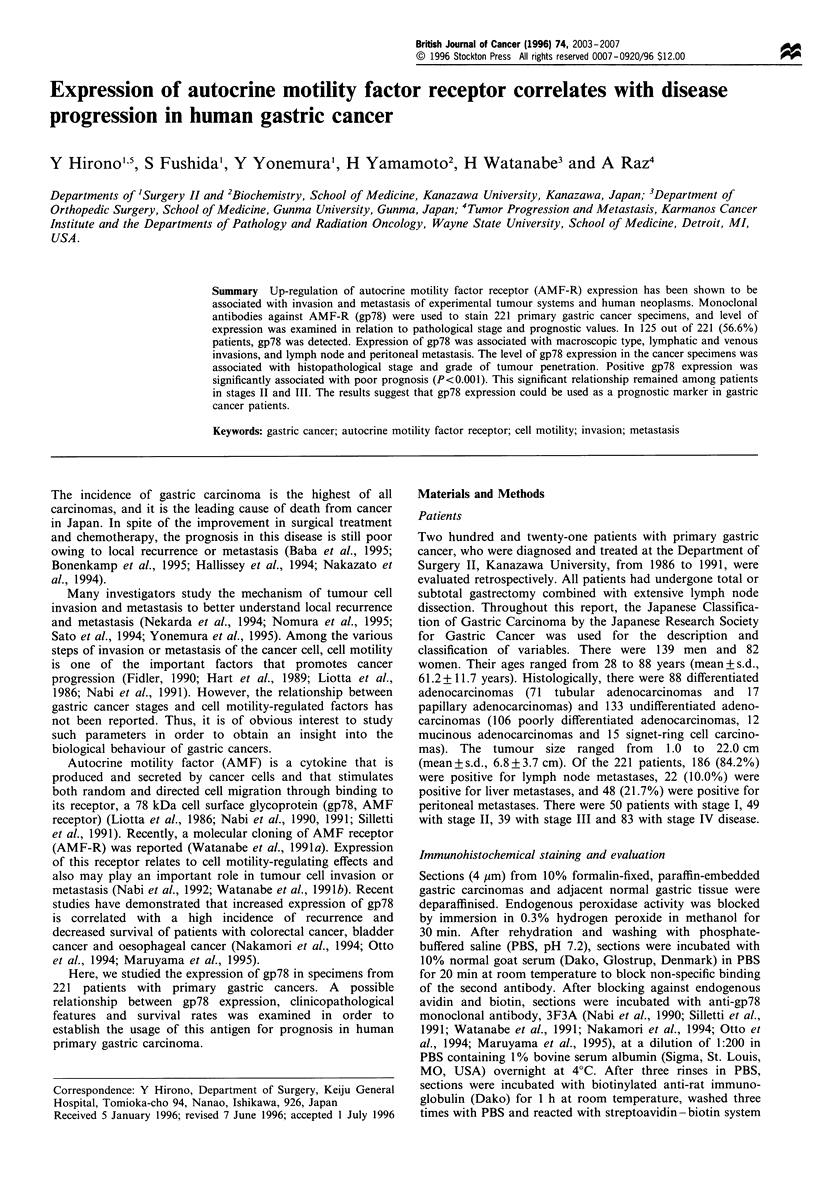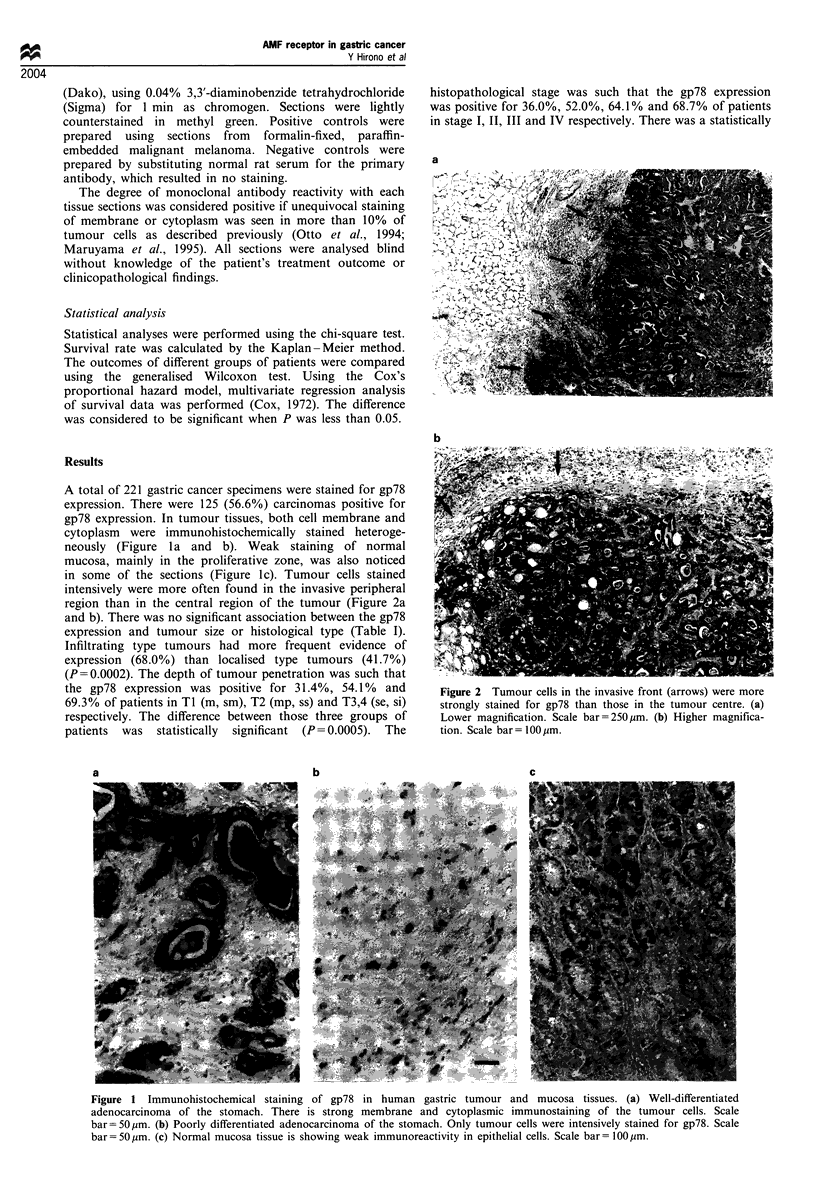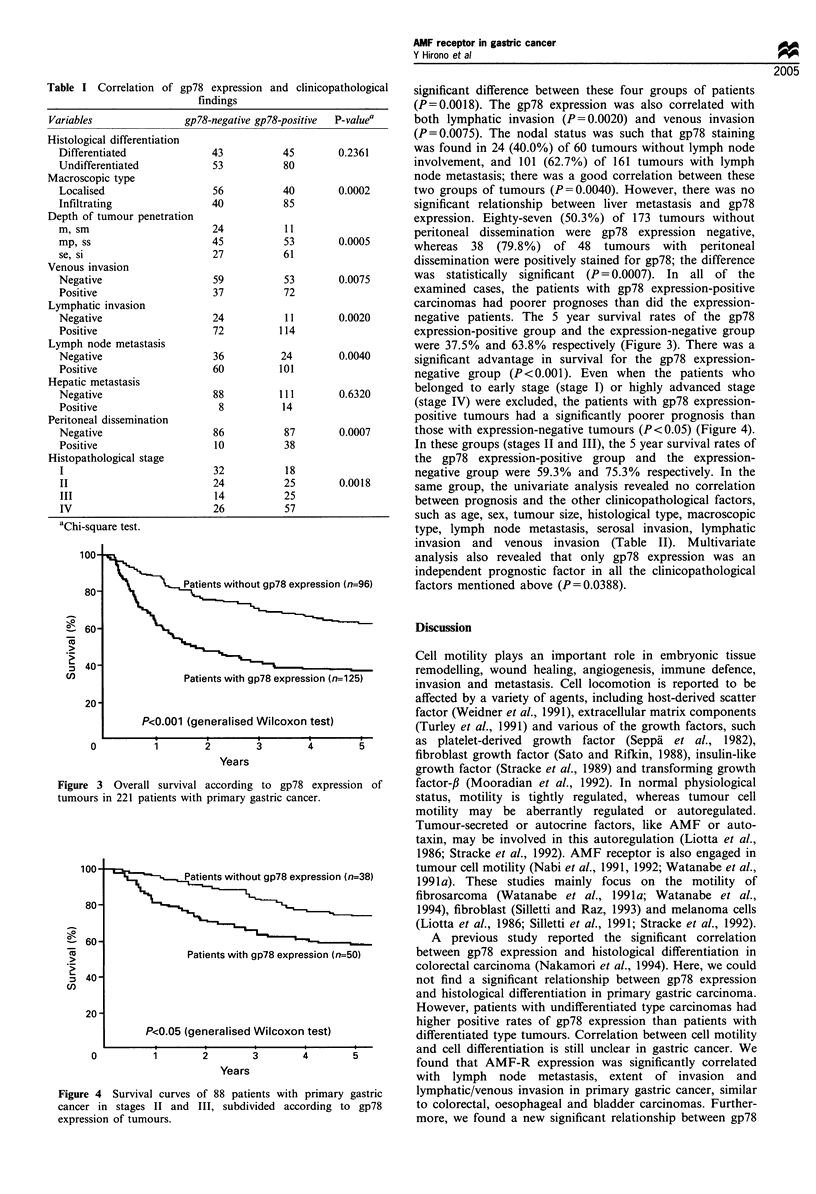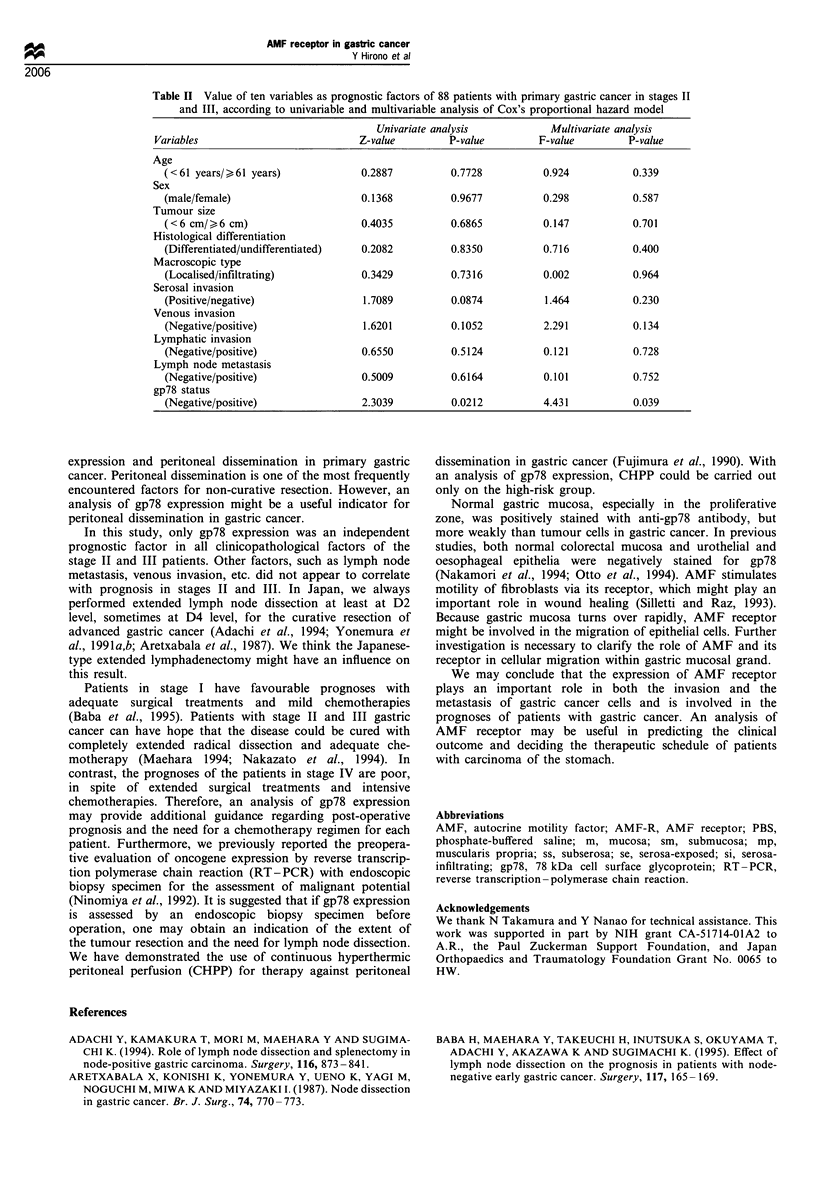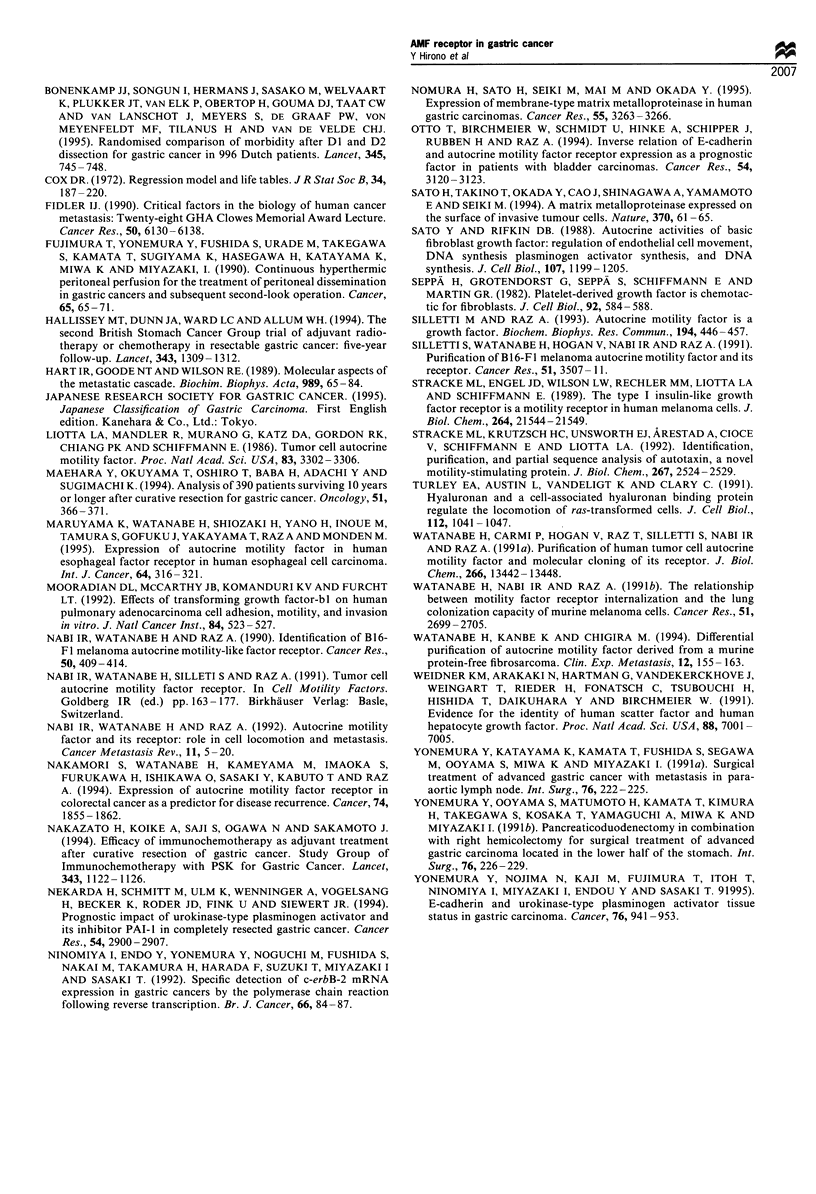# Expression of autocrine motility factor receptor correlates with disease progression in human gastric cancer.

**DOI:** 10.1038/bjc.1996.667

**Published:** 1996-12

**Authors:** Y. Hirono, S. Fushida, Y. Yonemura, H. Yamamoto, H. Watanabe, A. Raz

**Affiliations:** Department of Surgery II, School of Medicine, Kanazawa University, Japan.

## Abstract

**Images:**


British Journal of Cancer (1996) 74, 2003-2007
© 1996 Stockton Press All rights reserved 0007-0920/96 $12.00

### Expression of autocrine motility factor receptor correlates with disease progression in human gastric cancer

Y Hirono[1,5], S Fushida[1], Y Yonemura[1], H Yamamoto[2], H Watanabe[3] and A Raz[4]

*Departments of [1]Surgery II and [2]Biochemistry, School of Medicine, Kanazawa University, Kanazawa, Japan; [3]Department of Orthopedic Surgery, School of Medicine, Gunma University, Gunma, Japan; [4]Tumor Progression and Metastasis, Karmanos Cancer Institute and the Departments of Pathology and Radiation Oncology, Wayne State University, School of Medicine, Detroit, MI, USA.*

**Summary** Up-regulation of autocrine motility factor receptor (AMF-R) expression has been shown to be associated with invasion and metastasis of experimental tumour systems and human neoplasms. Monoclonal antibodies against AMF-R (gp78) were used to stain 221 primary gastric cancer specimens, and level of expression was examined in relation to pathological stage and prognostic values. In 125 out of 221 (56.6%) patients, gp78 was detected. Expression of gp78 was associated with macroscopic type, lymphatic and venous invasions, and lymph node and peritoneal metastasis. The level of gp78 expression in the cancer specimens was associated with histopathological stage and grade of tumour penetration. Positive gp78 expression was significantly associated with poor prognosis ($P<0.001$). This significant relationship remained among patients in stages II and III. The results suggest that gp78 expression could be used as a prognostic marker in gastric cancer patients.

**Keywords:** gastric cancer; autocrine motility factor receptor; cell motility; invasion; metastasis

The incidence of gastric carcinoma is the highest of all carcinomas, and it is the leading cause of death from cancer in Japan. In spite of the improvement in surgical treatment and chemotherapy, the prognosis in this disease is still poor owing to local recurrence or metastasis (Baba *et al.*, 1995; Bonenkamp *et al.*, 1995; Hallissey *et al.*, 1994; Nakazato *et al.*, 1994).

Many investigators study the mechanism of tumour cell invasion and metastasis to better understand local recurrence and metastasis (Nekarda *et al.*, 1994; Nomura *et al.*, 1995; Sato *et al.*, 1994; Yonemura *et al.*, 1995). Among the various steps of invasion or metastasis of the cancer cell, cell motility is one of the important factors that promotes cancer progression (Fidler, 1990; Hart *et al.*, 1989; Liotta *et al.*, 1986; Nabi *et al.*, 1991). However, the relationship between gastric cancer stages and cell motility-regulated factors has not been reported. Thus, it is of obvious interest to study such parameters in order to obtain an insight into the biological behaviour of gastric cancers.

Autocrine motility factor (AMF) is a cytokine that is produced and secreted by cancer cells and that stimulates both random and directed cell migration through binding to its receptor, a 78 kDa cell surface glycoprotein (gp78, AMF receptor) (Liotta *et al.*, 1986; Nabi *et al.*, 1990, 1991; Silletti *et al.*, 1991). Recently, a molecular cloning of AMF receptor (AMF-R) was reported (Watanabe *et al.*, 1991*a*). Expression of this receptor relates to cell motility-regulating effects and also may play an important role in tumour cell invasion or metastasis (Nabi *et al.*, 1992; Watanabe *et al.*, 1991*b*). Recent studies have demonstrated that increased expression of gp78 is correlated with a high incidence of recurrence and decreased survival of patients with colorectal cancer, bladder cancer and oesophageal cancer (Nakamori *et al.*, 1994; Otto *et al.*, 1994; Maruyama *et al.*, 1995).

Here, we studied the expression of gp78 in specimens from 221 patients with primary gastric cancers. A possible relationship between gp78 expression, clinicopathological features and survival rates was examined in order to establish the usage of this antigen for prognosis in human primary gastric carcinoma.

Correspondence: Y Hirono, Department of Surgery, Keiju General Hospital, Tomioka-cho 94, Nanao, Ishikawa, 926, Japan
Received 5 January 1996; revised 7 June 1996; accepted 1 July 1996

### Materials and Methods

### *Patients*

Two hundred and twenty-one patients with primary gastric cancer, who were diagnosed and treated at the Department of Surgery II, Kanazawa University, from 1986 to 1991, were evaluated retrospectively. All patients had undergone total or subtotal gastrectomy combined with extensive lymph node dissection. Throughout this report, the Japanese Classification of Gastric Carcinoma by the Japanese Research Society for Gastric Cancer was used for the description and classification of variables. There were 139 men and 82 women. Their ages ranged from 28 to 88 years (mean ± s.d., 61.2 ± 11.7 years). Histologically, there were 88 differentiated adenocarcinomas (71 tubular adenocarcinomas and 17 papillary adenocarcinomas) and 133 undifferentiated adenocarcinomas (106 poorly differentiated adenocarcinomas, 12 mucinous adenocarcinomas and 15 signet-ring cell carcinomas). The tumour size ranged from 1.0 to 22.0 cm (mean ± s.d., 6.8 ± 3.7 cm). Of the 221 patients, 186 (84.2%) were positive for lymph node metastases, 22 (10.0%) were positive for liver metastases, and 48 (21.7%) were positive for peritoneal metastases. There were 50 patients with stage I, 49 with stage II, 39 with stage III and 83 with stage IV disease.

### *Immunohistochemical staining and evaluation*

Sections (4 μm) from 10% formalin-fixed, paraffin-embedded gastric carcinomas and adjacent normal gastric tissue were deparaffinised. Endogenous peroxidase activity was blocked by immersion in 0.3% hydrogen peroxide in methanol for 30 min. After rehydration and washing with phosphate-buffered saline (PBS, pH 7.2), sections were incubated with 10% normal goat serum (Dako, Glostrup, Denmark) in PBS for 20 min at room temperature to block non-specific binding of the second antibody. After blocking against endogenous avidin and biotin, sections were incubated with anti-gp78 monoclonal antibody, 3F3A (Nabi *et al.*, 1990; Silletti *et al.*, 1991; Watanabe *et al.*, 1991; Nakamori *et al.*, 1994; Otto *et al.*, 1994; Maruyama *et al.*, 1995), at a dilution of 1:200 in PBS containing 1% bovine serum albumin (Sigma, St. Louis, MO, USA) overnight at 4°C. After three rinses in PBS, sections were incubated with biotinylated anti-rat immunoglobulin (Dako) for 1 h at room temperature, washed three times with PBS and reacted with streptoavidin–biotin system

(Dako), using 0.04% 3,3′-diaminobenzide tetrahydrochloride (Sigma) for 1 min as chromogen. Sections were lightly counterstained in methyl green. Positive controls were prepared using sections from formalin-fixed, paraffin-embedded malignant melanoma. Negative controls were prepared by substituting normal rat serum for the primary antibody, which resulted in no staining.

The degree of monoclonal antibody reactivity with each tissue sections was considered positive if unequivocal staining of membrane or cytoplasm was seen in more than 10% of tumour cells as described previously (Otto *et al.*, 1994; Maruyama *et al.*, 1995). All sections were analysed blind without knowledge of the patient's treatment outcome or clinicopathological findings.

### *Statistical analysis*

Statistical analyses were performed using the chi-square test. Survival rate was calculated by the Kaplan–Meier method. The outcomes of different groups of patients were compared using the generalised Wilcoxon test. Using the Cox's proportional hazard model, multivariate regression analysis of survival data was performed (Cox, 1972). The difference was considered to be significant when $P$ was less than 0.05.

### Results

A total of 221 gastric cancer specimens were stained for gp78 expression. There were 125 (56.6%) carcinomas positive for gp78 expression. In tumour tissues, both cell membrane and cytoplasm were immunohistochemically stained heterogeneously (Figure 1a and b). Weak staining of normal mucosa, mainly in the proliferative zone, was also noticed in some of the sections (Figure 1c). Tumour cells stained intensively were more often found in the invasive peripheral region than in the central region of the tumour (Figure 2a and b). There was no significant association between the gp78 expression and tumour size or histological type (Table I). Infiltrating type tumours had more frequent evidence of expression (68.0%) than localised type tumours (41.7%) ($P=0.0002$). The depth of tumour penetration was such that the gp78 expression was positive for 31.4%, 54.1% and 69.3% of patients in T1 (m, sm), T2 (mp, ss) and T3,4 (se, si) respectively. The difference between those three groups of patients was statistically significant ($P=0.0005$). The histopathological stage was such that the gp78 expression was positive for 36.0%, 52.0%, 64.1% and 68.7% of patients in stage I, II, III and IV respectively. There was a statistically

**Figure 2** Tumour cells in the invasive front (arrows) were more strongly stained for gp78 than those in the tumour centre. (**a**) Lower magnification. Scale bar = 250 $\mu$m. (**b**) Higher magnification. Scale bar = 100 $\mu$m.

**Figure 1** Immunohistochemical staining of gp78 in human gastric tumour and mucosa tissues. (**a**) Well-differentiated adenocarcinoma of the stomach. There is strong membrane and cytoplasmic immunostaining of the tumour cells. Scale bar = 50 $\mu$m. (**b**) Poorly differentiated adenocarcinoma of the stomach. Only tumour cells were intensively stained for gp78. Scale bar = 50 $\mu$m. (**c**) Normal mucosa tissue is showing weak immunoreactivity in epithelial cells. Scale bar = 100 $\mu$m.

Table I Correlation of gp78 expression and clinicopathological findings

| *Variables* | *gp78-negative* | *gp78-positive* | P-*value*[a] |
|---|---|---|---|
| Histological differentiation | | | |
| Differentiated | 43 | 45 | 0.2361 |
| Undifferentiated | 53 | 80 | |
| Macroscopic type | | | |
| Localised | 56 | 40 | 0.0002 |
| Infiltrating | 40 | 85 | |
| Depth of tumour penetration | | | |
| m, sm | 24 | 11 | |
| mp, ss | 45 | 53 | 0.0005 |
| se, si | 27 | 61 | |
| Venous invasion | | | |
| Negative | 59 | 53 | 0.0075 |
| Positive | 37 | 72 | |
| Lymphatic invasion | | | |
| Negative | 24 | 11 | 0.0020 |
| Positive | 72 | 114 | |
| Lymph node metastasis | | | |
| Negative | 36 | 24 | 0.0040 |
| Positive | 60 | 101 | |
| Hepatic metastasis | | | |
| Negative | 88 | 111 | 0.6320 |
| Positive | 8 | 14 | |
| Peritoneal dissemination | | | |
| Negative | 86 | 87 | 0.0007 |
| Positive | 10 | 38 | |
| Histopathological stage | | | |
| I | 32 | 18 | |
| II | 24 | 25 | 0.0018 |
| III | 14 | 25 | |
| IV | 26 | 57 | |

[a]Chi-square test.

Figure 3 Overall survival according to gp78 expression of tumours in 221 patients with primary gastric cancer.

Figure 4 Survival curves of 88 patients with primary gastric cancer in stages II and III, subdivided according to gp78 expression of tumours.

significant difference between these four groups of patients ($P = 0.0018$). The gp78 expression was also correlated with both lymphatic invasion ($P = 0.0020$) and venous invasion ($P = 0.0075$). The nodal status was such that gp78 staining was found in 24 (40.0%) of 60 tumours without lymph node involvement, and 101 (62.7%) of 161 tumours with lymph node metastasis; there was a good correlation between these two groups of tumours ($P = 0.0040$). However, there was no significant relationship between liver metastasis and gp78 expression. Eighty-seven (50.3%) of 173 tumours without peritoneal dissemination were gp78 expression negative, whereas 38 (79.8%) of 48 tumours with peritoneal dissemination were positively stained for gp78; the difference was statistically significant ($P = 0.0007$). In all of the examined cases, the patients with gp78 expression-positive carcinomas had poorer prognoses than did the expression-negative patients. The 5 year survival rates of the gp78 expression-positive group and the expression-negative group were 37.5% and 63.8% respectively (Figure 3). There was a significant advantage in survival for the gp78 expression-negative group ($P < 0.001$). Even when the patients who belonged to early stage (stage I) or highly advanced stage (stage IV) were excluded, the patients with gp78 expression-positive tumours had a significantly poorer prognosis than those with expression-negative tumours ($P < 0.05$) (Figure 4). In these groups (stages II and III), the 5 year survival rates of the gp78 expression-positive group and the expression-negative group were 59.3% and 75.3% respectively. In the same group, the univariate analysis revealed no correlation between prognosis and the other clinicopathological factors, such as age, sex, tumour size, histological type, macroscopic type, lymph node metastasis, serosal invasion, lymphatic invasion and venous invasion (Table II). Multivariate analysis also revealed that only gp78 expression was an independent prognostic factor in all the clinicopathological factors mentioned above ($P = 0.0388$).

### Discussion

Cell motility plays an important role in embryonic tissue remodelling, wound healing, angiogenesis, immune defence, invasion and metastasis. Cell locomotion is reported to be affected by a variety of agents, including host-derived scatter factor (Weidner *et al.*, 1991), extracellular matrix components (Turley *et al.*, 1991) and various of the growth factors, such as platelet-derived growth factor (Seppä *et al.*, 1982), fibroblast growth factor (Sato and Rifkin, 1988), insulin-like growth factor (Stracke *et al.*, 1989) and transforming growth factor-$\beta$ (Mooradian *et al.*, 1992). In normal physiological status, motility is tightly regulated, whereas tumour cell motility may be aberrantly regulated or autoregulated. Tumour-secreted or autocrine factors, like AMF or autotaxin, may be involved in this autoregulation (Liotta *et al.*, 1986; Stracke *et al.*, 1992). AMF receptor is also engaged in tumour cell motility (Nabi *et al.*, 1991, 1992; Watanabe *et al.*, 1991*a*). These studies mainly focus on the motility of fibrosarcoma (Watanabe *et al.*, 1991*a*; Watanabe *et al.*, 1994), fibroblast (Silletti and Raz, 1993) and melanoma cells (Liotta *et al.*, 1986; Silletti *et al.*, 1991; Stracke *et al.*, 1992).

A previous study reported the significant correlation between gp78 expression and histological differentiation in colorectal carcinoma (Nakamori *et al.*, 1994). Here, we could not find a significant relationship between gp78 expression and histological differentiation in primary gastric carcinoma. However, patients with undifferentiated type carcinomas had higher positive rates of gp78 expression than patients with differentiated type tumours. Correlation between cell motility and cell differentiation is still unclear in gastric cancer. We found that AMF-R expression was significantly correlated with lymph node metastasis, extent of invasion and lymphatic/venous invasion in primary gastric cancer, similar to colorectal, oesophageal and bladder carcinomas. Furthermore, we found a new significant relationship between gp78

**Table II** Value of ten variables as prognostic factors of 88 patients with primary gastric cancer in stages II and III, according to univariable and multivariable analysis of Cox's proportional hazard model

| *Variables* | *Univariate analysis* Z-*value* | P-*value* | *Multivariate analysis* F-*value* | P-*value* |
|---|---|---|---|---|
| Age | | | | |
| (<61 years/≥61 years) | 0.2887 | 0.7728 | 0.924 | 0.339 |
| Sex | | | | |
| (male/female) | 0.1368 | 0.9677 | 0.298 | 0.587 |
| Tumour size | | | | |
| (<6 cm/≥6 cm) | 0.4035 | 0.6865 | 0.147 | 0.701 |
| Histological differentiation | | | | |
| (Differentiated/undifferentiated) | 0.2082 | 0.8350 | 0.716 | 0.400 |
| Macroscopic type | | | | |
| (Localised/infiltrating) | 0.3429 | 0.7316 | 0.002 | 0.964 |
| Serosal invasion | | | | |
| (Positive/negative) | 1.7089 | 0.0874 | 1.464 | 0.230 |
| Venous invasion | | | | |
| (Negative/positive) | 1.6201 | 0.1052 | 2.291 | 0.134 |
| Lymphatic invasion | | | | |
| (Negative/positive) | 0.6550 | 0.5124 | 0.121 | 0.728 |
| Lymph node metastasis | | | | |
| (Negative/positive) | 0.5009 | 0.6164 | 0.101 | 0.752 |
| gp78 status | | | | |
| (Negative/positive) | 2.3039 | 0.0212 | 4.431 | 0.039 |

expression and peritoneal dissemination in primary gastric cancer. Peritoneal dissemination is one of the most frequently encountered factors for non-curative resection. However, an analysis of gp78 expression might be a useful indicator for peritoneal dissemination in gastric cancer.

In this study, only gp78 expression was an independent prognostic factor in all clinicopathological factors of the stage II and III patients. Other factors, such as lymph node metastasis, venous invasion, etc. did not appear to correlate with prognosis in stages II and III. In Japan, we always performed extended lymph node dissection at least at D2 level, sometimes at D4 level, for the curative resection of advanced gastric cancer (Adachi *et al.*, 1994; Yonemura *et al.*, 1991*a,b*; Aretxabala *et al.*, 1987). We think the Japanese-type extended lymphadenectomy might have an influence on this result.

Patients in stage I have favourable prognoses with adequate surgical treatments and mild chemotherapies (Baba *et al.*, 1995). Patients with stage II and III gastric cancer can have hope that the disease could be cured with completely extended radical dissection and adequate chemotherapy (Maehara 1994; Nakazato *et al.*, 1994). In contrast, the prognoses of the patients in stage IV are poor, in spite of extended surgical treatments and intensive chemotherapies. Therefore, an analysis of gp78 expression may provide additional guidance regarding post-operative prognosis and the need for a chemotherapy regimen for each patient. Furthermore, we previously reported the preoperative evaluation of oncogene expression by reverse transcription polymerase chain reaction (RT–PCR) with endoscopic biopsy specimen for the assessment of malignant potential (Ninomiya *et al.*, 1992). It is suggested that if gp78 expression is assessed by an endoscopic biopsy specimen before operation, one may obtain an indication of the extent of the tumour resection and the need for lymph node dissection. We have demonstrated the use of continuous hyperthermic peritoneal perfusion (CHPP) for therapy against peritoneal dissemination in gastric cancer (Fujimura *et al.*, 1990). With an analysis of gp78 expression, CHPP could be carried out only on the high-risk group.

Normal gastric mucosa, especially in the proliferative zone, was positively stained with anti-gp78 antibody, but more weakly than tumour cells in gastric cancer. In previous studies, both normal colorectal mucosa and urothelial and oesophageal epithelia were negatively stained for gp78 (Nakamori *et al.*, 1994; Otto *et al.*, 1994). AMF stimulates motility of fibroblasts via its receptor, which might play an important role in wound healing (Silletti and Raz, 1993). Because gastric mucosa turns over rapidly, AMF receptor might be involved in the migration of epithelial cells. Further investigation is necessary to clarify the role of AMF and its receptor in cellular migration within gastric mucosal grand.

We may conclude that the expression of AMF receptor plays an important role in both the invasion and the metastasis of gastric cancer cells and is involved in the prognoses of patients with gastric cancer. An analysis of AMF receptor may be useful in predicting the clinical outcome and deciding the therapeutic schedule of patients with carcinoma of the stomach.

### Abbreviations

AMF, autocrine motility factor; AMF-R, AMF receptor; PBS, phosphate-buffered saline; m, mucosa; sm, submucosa; mp, muscularis propria; ss, subserosa; se, serosa-exposed; si, serosa-infiltrating; gp78, 78 kDa cell surface glycoprotein; RT–PCR, reverse transcription–polymerase chain reaction.

### Acknowledgements

We thank N Takamura and Y Nanao for technical assistance. This work was supported in part by NIH grant CA-51714-01A2 to A.R., the Paul Zuckerman Support Foundation, and Japan Orthopaedics and Traumatology Foundation Grant No. 0065 to HW.

### References

ADACHI Y, KAMAKURA T, MORI M, MAEHARA Y AND SUGIMACHI K. (1994). Role of lymph node dissection and splenectomy in node-positive gastric carcinoma. *Surgery*, **116**, 873–841.

ARETXABALA X, KONISHI K, YONEMURA Y, UENO K, YAGI M, NOGUCHI M, MIWA K AND MIYAZAKI I. (1987). Node dissection in gastric cancer. *Br. J. Surg.*, **74**, 770–773.

BABA H, MAEHARA Y, TAKEUCHI H, INUTSUKA S, OKUYAMA T, ADACHI Y, AKAZAWA K AND SUGIMACHI K. (1995). Effect of lymph node dissection on the prognosis in patients with node-negative early gastric cancer. *Surgery*, **117**, 165–169.

BONENKAMP JJ, SONGUN I, HERMANS J, SASAKO M, WELVAART K, PLUKKER JT, VAN ELK P, OBERTOP H, GOUMA DJ, TAAT CW AND VAN LANSCHOT J, MEYERS S, DE GRAAF PW, VON MEYENFELDT MF, TILANUS H AND VAN DE VELDE CHJ. (1995). Randomised comparison of morbidity after D1 and D2 dissection for gastric cancer in 996 Dutch patients. *Lancet*, **345,** 745–748.

COX DR. (1972). Regression model and life tables. *J R Stat Soc B*, **34,** 187–220.

FIDLER IJ. (1990). Critical factors in the biology of human cancer metastasis: Twenty-eight GHA Clowes Memorial Award Lecture. *Cancer Res.*, **50,** 6130–6138.

FUJIMURA T, YONEMURA Y, FUSHIDA S, URADE M, TAKEGAWA S, KAMATA T, SUGIYAMA K, HASEGAWA H, KATAYAMA K, MIWA K AND MIYAZAKI, I. (1990). Continuous hyperthermic peritoneal perfusion for the treatment of peritoneal dissemination in gastric cancers and subsequent second-look operation. *Cancer*, **65,** 65–71.

HALLISSEY MT, DUNN JA, WARD LC AND ALLUM WH. (1994). The second British Stomach Cancer Group trial of adjuvant radiotherapy or chemotherapy in resectable gastric cancer: five-year follow-up. *Lancet*, **343,** 1309–1312.

HART IR, GOODE NT AND WILSON RE. (1989). Molecular aspects of the metastatic cascade. *Biochim. Biophys. Acta*, **989,** 65–84.

JAPANESE RESEARCH SOCIETY FOR GASTRIC CANCER. (1995). *Japanese Classification of Gastric Carcinoma.* First English edition. Kanehara & Co., Ltd.: Tokyo.

LIOTTA LA, MANDLER R, MURANO G, KATZ DA, GORDON RK, CHIANG PK AND SCHIFFMANN E. (1986). Tumor cell autocrine motility factor. *Proc. Natl Acad. Sci. USA*, **83,** 3302–3306.

MAEHARA Y, OKUYAMA T, OSHIRO T, BABA H, ADACHI Y AND SUGIMACHI K. (1994). Analysis of 390 patients surviving 10 years or longer after curative resection for gastric cancer. *Oncology*, **51,** 366–371.

MARUYAMA K, WATANABE H, SHIOZAKI H, YANO H, INOUE M, TAMURA S, GOFUKU J, YAKAYAMA T, RAZ A AND MONDEN M. (1995). Expression of autocrine motility factor in human esophageal factor receptor in human esophageal cell carcinoma. *Int. J. Cancer*, **64,** 316–321.

MOORADIAN DL, McCARTHY JB, KOMANDURI KV AND FURCHT LT. (1992). Effects of transforming growth factor-b1 on human pulmonary adenocarcinoma cell adhesion, motility, and invasion *in vitro*. *J. Natl Cancer Inst.*, **84,** 523–527.

NABI IR, WATANABE H AND RAZ A. (1990). Identification of B16-F1 melanoma autocrine motility-like factor receptor. *Cancer Res.*, **50,** 409–414.

NABI IR, WATANABE H, SILLETI S AND RAZ A. (1991). Tumor cell autocrine motility factor receptor. In *Cell Motility Factors.* Goldberg IR (ed.) pp. 163–177. Birkhäuser Verlag: Basle, Switzerland.

NABI IR, WATANABE H AND RAZ A. (1992). Autocrine motility factor and its receptor: role in cell locomotion and metastasis. *Cancer Metastasis Rev.*, **11,** 5–20.

NAKAMORI S, WATANABE H, KAMEYAMA M, IMAOKA S, FURUKAWA H, ISHIKAWA O, SASAKI Y, KABUTO T AND RAZ A. (1994). Expression of autocrine motility factor receptor in colorectal cancer as a predictor for disease recurrence. *Cancer*, **74,** 1855–1862.

NAKAZATO H, KOIKE A, SAJI S, OGAWA N AND SAKAMOTO J. (1994). Efficacy of immunochemotherapy as adjuvant treatment after curative resection of gastric cancer. Study Group of Immunochemotherapy with PSK for Gastric Cancer. *Lancet*, **343,** 1122–1126.

NEKARDA H, SCHMITT M, ULM K, WENNINGER A, VOGELSANG H, BECKER K, RODER JD, FINK U AND SIEWERT JR. (1994). Prognostic impact of urokinase-type plasminogen activator and its inhibitor PAI-1 in completely resected gastric cancer. *Cancer Res.*, **54,** 2900–2907.

NINOMIYA I, ENDO Y, YONEMURA Y, NOGUCHI M, FUSHIDA S, NAKAI M, TAKAMURA H, HARADA F, SUZUKI T, MIYAZAKI I AND SASAKI T. (1992). Specific detection of c-*erb*B-2 mRNA expression in gastric cancers by the polymerase chain reaction following reverse transcription. *Br. J. Cancer*, **66,** 84–87.

NOMURA H, SATO H, SEIKI M, MAI M AND OKADA Y. (1995). Expression of membrane-type matrix metalloproteinase in human gastric carcinomas. *Cancer Res.*, **55,** 3263–3266.

OTTO T, BIRCHMEIER W, SCHMIDT U, HINKE A, SCHIPPER J, RUBBEN H AND RAZ A. (1994). Inverse relation of E-cadherin and autocrine motility factor receptor expression as a prognostic factor in patients with bladder carcinomas. *Cancer Res.*, **54,** 3120–3123.

SATO H, TAKINO T, OKADA Y, CAO J, SHINAGAWA A, YAMAMOTO E AND SEIKI M. (1994). A matrix metalloproteinase expressed on the surface of invasive tumour cells. *Nature*, **370,** 61–65.

SATO Y AND RIFKIN DB. (1988). Autocrine activities of basic fibroblast growth factor: regulation of endothelial cell movement, DNA synthesis plasminogen activator synthesis, and DNA synthesis. *J. Cell Biol.*, **107,** 1199–1205.

SEPPÄ H, GROTENDORST G, SEPPÄ S, SCHIFFMANN E AND MARTIN GR. (1982). Platelet-derived growth factor is chemotactic for fibroblasts. *J. Cell Biol.*, **92,** 584–588.

SILLETTI M AND RAZ A. (1993). Autocrine motility factor is a growth factor. *Biochem. Biophys. Res. Commun.*, **194,** 446–457.

SILLETTI S, WATANABE H, HOGAN V, NABI IR AND RAZ A. (1991). Purification of B16-F1 melanoma autocrine motility factor and its receptor. *Cancer Res.*, **51,** 3507–11.

STRACKE ML, ENGEL JD, WILSON LW, RECHLER MM, LIOTTA LA AND SCHIFFMANN E. (1989). The type I insulin-like growth factor receptor is a motility receptor in human melanoma cells. *J. Biol. Chem.*, **264,** 21544–21549.

STRACKE ML, KRUTZSCH HC, UNSWORTH EJ, ÅRESTAD A, CIOCE V, SCHIFFMANN E AND LIOTTA LA. (1992). Identification, purification, and partial sequence analysis of autotaxin, a novel motility-stimulating protein. *J. Biol. Chem.*, **267,** 2524–2529.

TURLEY EA, AUSTIN L, VANDELIGT K AND CLARY C. (1991). Hyaluronan and a cell-associated hyaluronan binding protein regulate the locomotion of *ras*-transformed cells. *J. Cell Biol.*, **112,** 1041–1047.

WATANABE H, CARMI P, HOGAN V, RAZ T, SILLETTI S, NABI IR AND RAZ A. (1991*a*). Purification of human tumor cell autocrine motility factor and molecular cloning of its receptor. *J. Biol. Chem.*, **266,** 13442–13448.

WATANABE H, NABI IR AND RAZ A. (1991*b*). The relationship between motility factor receptor internalization and the lung colonization capacity of murine melanoma cells. *Cancer Res.*, **51,** 2699–2705.

WATANABE H, KANBE K AND CHIGIRA M. (1994). Differential purification of autocrine motility factor derived from a murine protein-free fibrosarcoma. *Clin. Exp. Metastasis*, **12,** 155–163.

WEIDNER KM, ARAKAKI N, HARTMAN G, VANDEKERCKHOVE J, WEINGART T, RIEDER H, FONATSCH C, TSUBOUCHI H, HISHIDA T, DAIKUHARA Y AND BIRCHMEIER W. (1991). Evidence for the identity of human scatter factor and human hepatocyte growth factor. *Proc. Natl Acad. Sci. USA*, **88,** 7001–7005.

YONEMURA Y, KATAYAMA K, KAMATA T, FUSHIDA S, SEGAWA M, OOYAMA S, MIWA K AND MIYAZAKI I. (1991*a*). Surgical treatment of advanced gastric cancer with metastasis in para-aortic lymph node. *Int. Surg.*, **76,** 222–225.

YONEMURA Y, OOYAMA S, MATUMOTO H, KAMATA T, KIMURA H, TAKEGAWA S, KOSAKA T, YAMAGUCHI A, MIWA K AND MIYAZAKI I. (1991*b*). Pancreaticoduodenectomy in combination with right hemicolectomy for surgical treatment of advanced gastric carcinoma located in the lower half of the stomach. *Int. Surg.*, **76,** 226–229.

YONEMURA Y, NOJIMA N, KAJI M, FUJIMURA T, ITOH T, NINOMIYA I, MIYAZAKI I, ENDOU Y AND SASAKI T. 91995). E-cadherin and urokinase-type plasminogen activator tissue status in gastric carcinoma. *Cancer*, **76,** 941–953.